# Percutaneous Iliac Screws for Minimally Invasive Spinal Deformity Surgery

**DOI:** 10.1155/2012/173685

**Published:** 2012-07-29

**Authors:** Michael Y. Wang

**Affiliations:** Lois Pope Life Center, Department of Neurological Surgery, University of Miami Miller School of Medicine, 1095 NW 14th Terrace, Miami, FL 33136, USA

## Abstract

*Introduction*. Adult spinal deformity (ASD) surgeries carry significant morbidity, and this has led many surgeons to apply minimally invasive surgery (MIS) techniques to reduce the blood loss, infections, and other peri-operative complications. A spectrum of techniques for MIS correction of ASD has thus evolved, most recently the application of percutaneous iliac screws. *Methods*. Over an 18 months 10 patients with thoracolumbar scoliosis underwent MIS surgery. The mean age was 73 years (70% females). Patients were treated with multi-level facet osteotomies and interbody fusion using expandable cages followed by percutaneous screw fixation. Percutaneous iliac screws were placed bilaterally using the obturator outlet view to target the ischial body. *Results*. All patients were successfully instrumented without conversion to an open technique. Mean operative time was 302 minutes and the mean blood loss was 480 cc, with no intraoperative complications. A total of 20 screws were placed successfully as judged by CT scanning to confirm no bony violations. Complications included: two asymptomatic medial breaches at T10 and L5, and one patient requiring delayed epidural hematoma evacuation. *Conclusions*. Percutaneous iliac screws can be placed safely in patients with ASD. This MIS technique allows for successful caudal anchoring to stress-shield the sacrum and L5-S1 fusion site in long-segment constructs.

## 1. Introduction

Surgery to correct adult spinal deformity (ASD) is a growing field. The ever-aging American population is presenting to spinal surgeons increasingly with high expectations of continued quality of life well into the seventh, eighth, and ninth decades of life. However, while surgical treatment of ASD is the only viable option for patients failing conservative measures, the surgical interventions are associated with relatively high morbidity and mortality rates. Indeed, in a series reported from Johns' Hopkins consisting of 361 patients, the 30-day mortality rate was found to be 2.4% [1]. In a more series by Smith et al., multicenter data from the Spinal Deformity Study Group demonstrated that even in expert centers 26.2% of patients suffered a minor complication and 15.5% suffered a major complication [2].

 Several factors contribute to these high complication rates, including reduced bone mass and weaker fixation points, a higher associated rate of medical comorbidites, patient deconditioning due to immobility, and a rigid and nonflexible deformity [3, 4]. In addition, the surgical enterprise necessary to correct ASD is typically a long-segment fusion with instrumentation and osteotomies. Therefore, in this population, a major surgical intervention is being applied in a highly compromised patient population [5, 6]. 

 To combat these challenges, modern surgeons have begun to apply minimally invasive surgery (MIS) techniques to address ASD [7–9]. MIS techniques have been associated with reduced intraoperative blood loss, lower infection rates, and quicker mobilization, all of which would be highly desirable in the ASD population [10]. While the early MIS fusion experience has focused on one- and two-level procedures for degenerative spinal disease, a variety of techniques have been developed more recently for use in ASD. 

 One major advance in spinal fixation has been the application of iliac fixation. Pelvic fixation is an important tool in the armamentarium of the modern spinal surgeon, as screws or bolts of a large diameter and length can be placed safely for caudal anchoring and extend anterior to the spine in the sagittal plane and lateral to it in the coronal plane. Iliac fixation is useful in ASD for long instrumentation constructs, sagittal and coronal deformity corrections, and stabilization of low sacropelvic instability [11–13].

We previously published a technique for percutaneous iliac screw fixation [14]. This paper builds upon that experience with the application of this technique in the setting of ASD.

## 2. Methods

### 2.1. Patient Population

 A consecutive series of 10 patients were treated over an 18-month period at a single institution. All patients underwent MIS treatment of ASD using expandable interbody cage placement and percutaneous pedicle and iliac screws. ASD was defined as a Cobb angle greater than 20°. All deformities were rigid with less than 10° of motion in the coronal or sagittal planes across the deformity segments on flexion, extension, and lateral bending films. All patients had also failed conservative measures and had severe back and/or back and leg pain with distance limited gait. The accuracy of iliac screw insertion was examined using postoperative spiral CT scanning to confirm that screws were entirely within the bony confines. 

### 2.2. Surgical Technique

**Figure 1:**
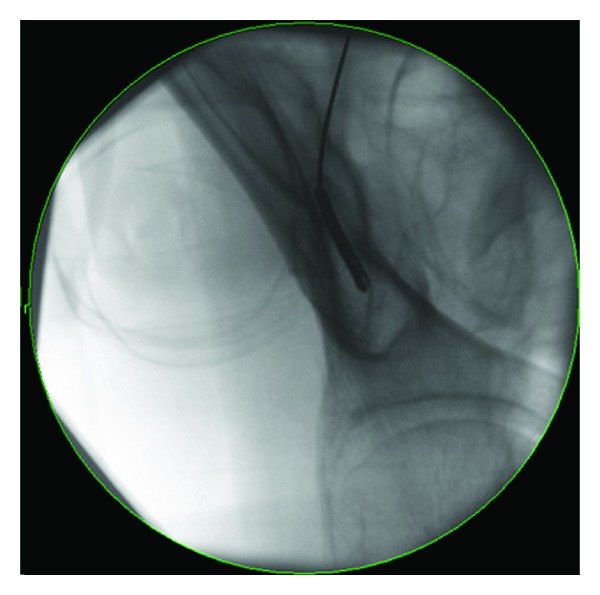
Obturator outlet view showing the “teardrop” target for iliac screw placement. Cannulation of this space provides a safe corridor completely within the bony confines.

**Figure 2:**
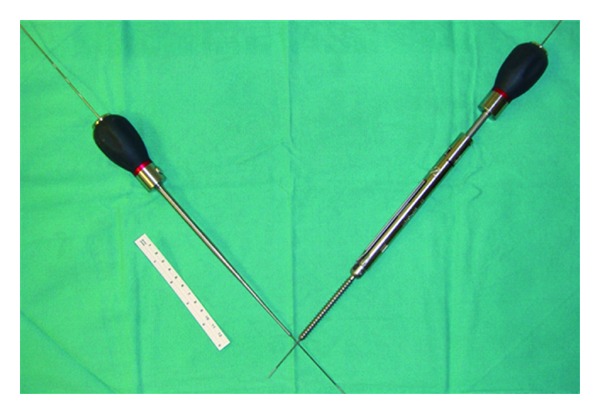
Cannulated 8 mm diameter by 80 mm long screws for iliac fixation and cannulated cancellous bone probe.

Patients were positioned prone on the Jackson table so that the pelvis would not be obscured on fluoroscopic imaging by the base of the operating table. Pre-operative imaging, including 3 D reconstructed CT scans of the pelvis, was helpful for planning screw placement trajectories and to validate the fluoroscopic data in the operating room. Iliac cannulation is performed prior to pedicle screw cannulation to maximize the ability to image the pelvis. In addition, the decompression, osteotomies, and interbody fusion are accomplished prior to screw placement. For each side of the iliac crest, the fluoroscope is angled in the sagittal and coronal planes in the obturator outlet view so that the X-ray beams are approximately parallel to both the inner and outer tables of the ilium (Figure 1). The “teardrop” that is visualized is the safe corridor and placement of instrumentation within this two-dimensional space ensures safe screw placement, even with 80 mm long screws (Figure 2). 

**Figure 3:**
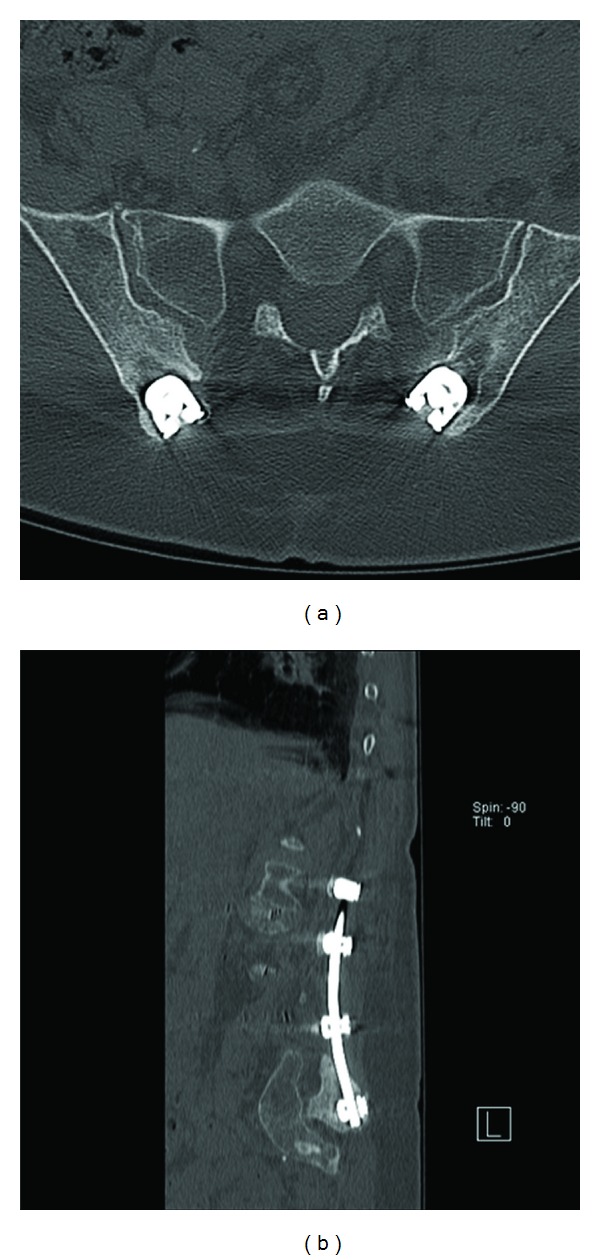
Recession of the iliac screw saddles into the bone to avoid hardware prominence as seen on this postoperative (a) axial and (b) sagittal reconstruction CT scan.

A 1.5 cm incision is then made overlying the posterior superior iliac spine of the pelvis (PSIS). A Jamshidi needle is then docked onto the most superficial aspect of the PSIS and “walked” ventromedially, with care not to enter into the sacroiliac joint. However, the exact starting point along the superinferior plane of the PSIS can vary according to the specific screw trajectory desired, as multiple acceptable paths are acceptable. A drill or osteotome can be used to create a bony depression to better seat the screw or bolt head to minimize hardware prominence (Figure 3). After entering 55–75 mm, the Jamshidi needle is then replaced internally with a K-wire and then removed. Cannulated cancellous screw taps are then placed over the K-wire followed by final screw insertion.

**Figure 4:**
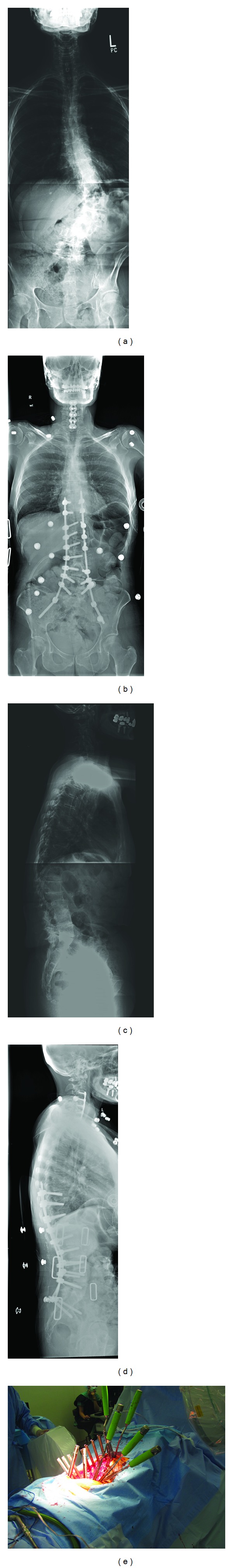
Case example showing a T9 to Iliac MIS fusion with interbody grafts at L2-S1. (a) and (b) Pre- and postoperative AP, and (c) and (d) Pre- and postoperative lateral 36” X-Ray images. (e) Intraoperative view.

**Figure 5:**
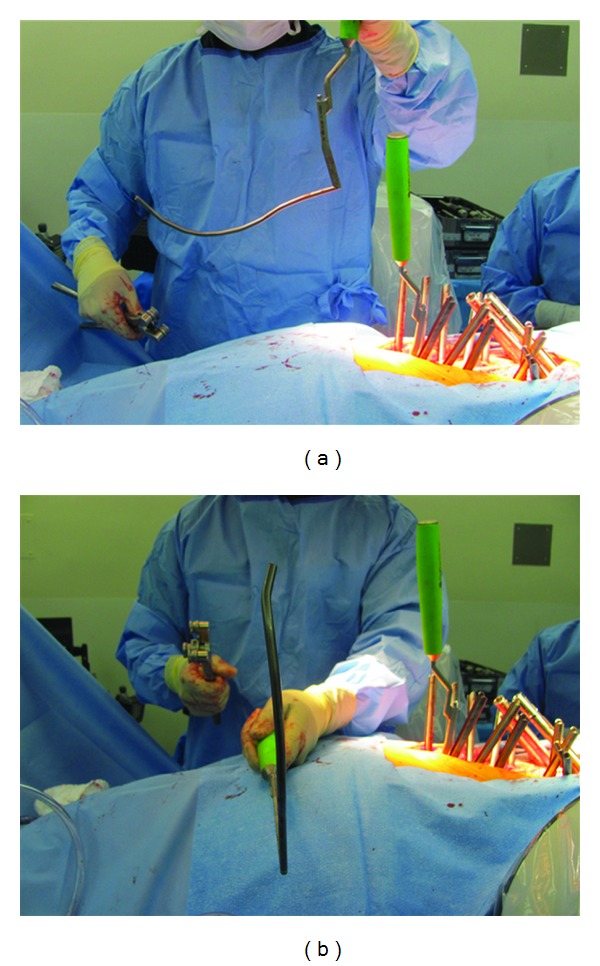
Two plane rods bending in the (a) sagittal and (b) coronal planes to facilitate connection to the more laterally located iliac screw saddles.

Pedicle screw cannulation and placement then proceed followed by rod insertion and hookup (Figure 4). Since the iliac screws will be more dorsal and lateral than pedicle screws, the appropriate rod bending in two planes facilitates screw-rod mating. In addition, starting the S1 screws high and the iliac screws low provides more distance between the screw heads, making the connection easier (Figure 5). Bending the rods while attached to the rod holder facilitates this two-plane bending when using a French bender. The exact amount of curvature to place in the rods is based upon the surgeon's judgement of preoperative curvature, desired degree of correction, and flexibility in the spine after decompression and osteotomies.

## 3. Results

 The series was consecutive with no patients lost to followup, and in no case was conversion to a traditional open technique necessary. A total of 10 patients (7 women and 3 men) were treated using this technique (Table 1). Their mean age was 73 years, with a range of 62 to 80. The average BMI was 28. A total of 69 segmental levels were treated (mean = 6.9), with a range of 4–9. A total of 20 percutaneous iliac screws were placed. The mean operative time was 302 minutes from skin-to-skin, and the mean intraoperative blood loss as measured by the perfusionist was 480 cc. Length of acute care stay averaged 5.6 days (range of 4–7) after surgery. Three of the 10 patients were discharged to an inpatient rehabilitation facility, and the rest were discharged to home. 65 mm × 8 mm screws were used in 5 patients, and 80 mm × 8 mm screws were used in 5 patients. All patients had interbody allograft cages placed at the L5/S1 level.

 Early radiographic outcomes were determined using pre-and postoperative 36” standing X-rays at last followup. The mean preoperative Cobb angle was 35° which improved to a mean of 8.0°, reflecting an average of 27° of improvement. The mean preoperative global lumbar lordosis as measured between L1 and S1 was 27° which improved to a mean of 48°, reflecting an average of 21° of improvement. All 20 iliac screws were placed successfully as judged by postoperative CT scanning. 

 There were no intraoperative complications. However, one patient had two asymptomatic medial screw breaches at T10 and L5. This patient did not undergo reoperation as there was no neurological impairment. A second patient developed a symptomatic epidural hematoma on postoperative day number 6. This was evacuated emergently with neurological recovery. 

## 4. Discussion

 Due to the many benefits of MIS surgery, it has the potential to improve the outcomes of surgery for ASD. Because these patients are often medically compromised, a reduction in infection rates, intraoperative blood loss, and quicker mobilization may have a significant impact on their recovery. While in the past MIS surgeons focused primarily on short segment fusions for degenerative disease [10], there is increasing interest in using MIS techniques for ASD. However, the concept that is emerging for MIS deformity surgery is that the goals and standards being developed for open deformity surgery must also be met with MIS surgery.

 In this paper we describe our initial experience with percutaneous iliac screws for treating ASD. While the series is of limited size, radiographic evaluation demonstrated safe iliac screw placement using a relatively straightforward technique that did not require specialized equipment is possible. Using a single C-arm and the obturator outlet view, standard size iliac screws could be placed safely and efficiently. While image guidance can be helpful in many settings, navigation systems are expensive, prone to error, and require additional setup time. Thus, we have chosen to continue using a simplified C-arm method for screw placement. The introduction of commercially available cannulated iliac screws has also helped to make this procedure widely accessible to surgeons and renders the procedure as accessible as open screw placement. It should however be remembered that screw misplacement with any surgical technique can result in sciatic nerve injury, major vessel disruption, pelvic fracture, or retroperitoneal hematoma formation, and these risks are higher in the ASD population.

 When applying this technique, many of the considerations for open surgery are relevant to the MIS setting. For example, strict attention needs to be placed to screw head positioning. It is critical to recess the iliac screw heads to reduce complaints of hardware prominence. This can be accomplished by using the drill or osteotome to created an opening in the posterior cortical wall of the ilium. In additional, starting the screw below the PSIS keeps the saddle low. With regard to hardware connections, placing the iliac screw heads medial and the pedicle screws lateral keeps the screw saddles in a single plane and facilitates rod-screw mating. However, despite these efforts, multiple-rod plane bending is often necessary as lateral offset connectors cannot be applied using a truly percutaneous method. 

 It should also be noted that in this series the screws were either 65 or 80 mm in length. Open deformity surgeons commonly use longer screws to obtain superior fixation. In this series, we generally did not treat cases of severe scoliosis (>60°) or major kyphosis, and the series also did not include serious revisions and thus have had success with the shorter iliac screws. Furthermore, maintenance of the soft tissue envelope and posterior tension band with MIS surgery preserves the spine's native integrity and thus may obviate the need for these longer screws. Ultimately, the placement of screws greater than 100 mm in length should be feasible but will be yet another area requiring validation in the clinical setting.

 While MIS surgery for ASD has not been able to completely replace open, conventional methods, the expanding spectrum of MIS techniques has allowed the modern MIS surgeon to perform ever more complex surgeries in this patient population. Percutaneous iliac screws represent one such advance to allow for successful caudal anchoring of long-segment spinal fixation constructs. 

## Figures and Tables

**Table 1:** 

Patient	Sex	Age	Operative procedure	Complications	Time	EBL	LOS	Dispo	Iliac screws	CT confirmation of iliac screw placement
JB	M	68	L1-iliac MIS instrumented fusion with L2-S1 MIS TLIF	None	360	800	4	Home	65 mm × 8 mm	Yes, correct position
DK	F	75	T10-iliac MIS instrumented fusion with L2-S1 MIS TLIF	None	320	500	5	Home	80 mm × 8 mm	Yes, correct position
HS	F	78	T9-iliac MIS instrumented fusion with L1-S1 MIS TLIF	None	340	550	7	Home	65 mm × 8 mm	Yes, correct position
JL	F	72	T9-iliac MIS instrumented fusion with L1–5 MIS TLIF	None	300	450	6	Home	80 mm × 8 mm	Yes, correct position
KF	F	62	T11-iliac MIS instrumented fusion with L5-S1 MIS TLIF	None	265	250	5	Home	65 mm × 8 mm	Yes, correct position
ES	F	76	T10-iliac MIS instrumented fusion with L2-S1 MSI TLIF	T10 and L5 screw breaches	310	500	8	Rehab	80 mm × 8 mm	Yes, correct position
RS	F	75	T12-iliac MIS instrumented fusion with L2-S1 MSI TLIF	None	310	400	4	Rehab	65 mm × 8 mm	Yes, correct position
BP	M	80	L2-iliac MIS instrumented fusion with L2-S1 MIS TLIF	None	280	450	5	Home	80 mm × 8 mm	Yes, correct position
SR	F	66	T9-iliac MIS instrumented fusion with L1–5 MIS TLIF	Epidural hematoma requiring laminectomy for evacuation	300	500	7	Rehab	65 mm × 8 mm	Yes, correct position
RL	M	78	L2-iliac MIS instrumented fusion with L2-S1 MIS TLIF	None	240	395	5	Home	80 mm × 8 mm	Yes, correct position
